# Piezochromic Phenomena of Nanometer Voids Formed by Mono-Dispersed Nanometer Powders Compacting Process

**DOI:** 10.1371/journal.pone.0072964

**Published:** 2013-10-07

**Authors:** Lihong Su, Caixia Wan, Jianren Zhou, Yiguang Wang, Liang Wang, Yanling Ai, Xu Zhao

**Affiliations:** 1 Department of Applying Chemistry, Northwestern Polytechnical University, Xi'An, Shaanxi Province, China; 2 College of Engineering, Prairie View A&M University, Prairie View, Texas, United States of America; RMIT University, Australia

## Abstract

Piezochromism describes a tendency of certain materials changing colors when they are subjected to various pressure levels. It occurs particularly in some polymers or inorganic materials, such as in palladium complexes. However, piezochromism is generally believed to work at high pressure range of 0.1–10 GPa. This research work focused on unique piezochromism responses of the nanometer voids formed by the 5–20 nm inorganic ISOH nanometer powders. It was discovered that microstructures of the nanometer voids could change color at very low pressures of only 0.002–0.01 GPa; its sensitivity to pressure was increased by tens of times. It is believed that the uniform microstructures of nanometer powders contributed to the material's high sensitivity of piezochromic phenomena. One factor which quantum optical change caused by nanometer voids affected the quantum confinement effect; another is surface Plasmon Resonance of great difference dielectric property between conductive ITO powder and insulation hydroxide.

## Introduction

Piezochromic materials can change their colors as they are under a pressure, which makes them attractive and has many potential applications in industries and our everyday life. The piezochromic effects were found originally in some polymers and polymers with metal-complexes. The piezochromic materials can be divided into three common types: organic molecules, metal cluster compounds, and organic complexes [1]–[10]. The pressure sensitivity and temperature tolerance of these materials are generally poor: only work well at high pressures (usually at 0.1–10 GPa) and relatively low temperatures (below 150°C) [11]–[16]. It has been well known that some nanometer particles can undergo colour change as their sizes change due to the fact that the particle dimensions are in the proximity or far small wavelengths of the visible rays [7] [9].

This work investigated the piezochromic mechanism of nanometer voids formed by 5–20 nm inorganic nanometer powder mixtures. It was found that these materials can change their colours at a low pressure range of 0.002–0.1 GPa. The piezochromic sensitivity of the nanometer voids was increased tens of times as compared with the traditional polymeric materials. In addition, the piezochromic process was reversible; the colors change back to prior ones as the pressures was released and milled to powders again. The nanometer material also has a better temperature tolerance than the traditional polymeric materials. Because the piezochromic phenomena can easily generate phonetics-electronic signals and the color changing can be related to the time in a slow-pace event, it will be an ideal material for forecast of long-range events, such as mud-rock flow, landslide, or avalanche geological hazards, it does not depend on power source and index pressure directly, it is effective and simple early warning material that can detect only 1 mm displacement of bridge or rock, thus it would help the trackwalker to increase the high-speed rail safety in China. The material is insoluble in water or organic solvent. It can be applied in the cell expansibility detection when it placed between two flexible transparent films at closed glass tube, and differentiated the pathological changes of cell by the different color index.

## Experimental

### 1. Material preparation

**Figure 1:**
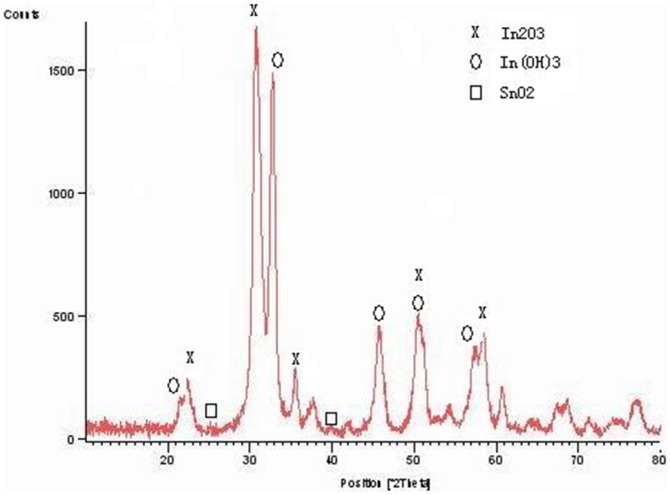
The XRD pattern of 5–20 nm nanometer powder mixture.

**Figure 2:**
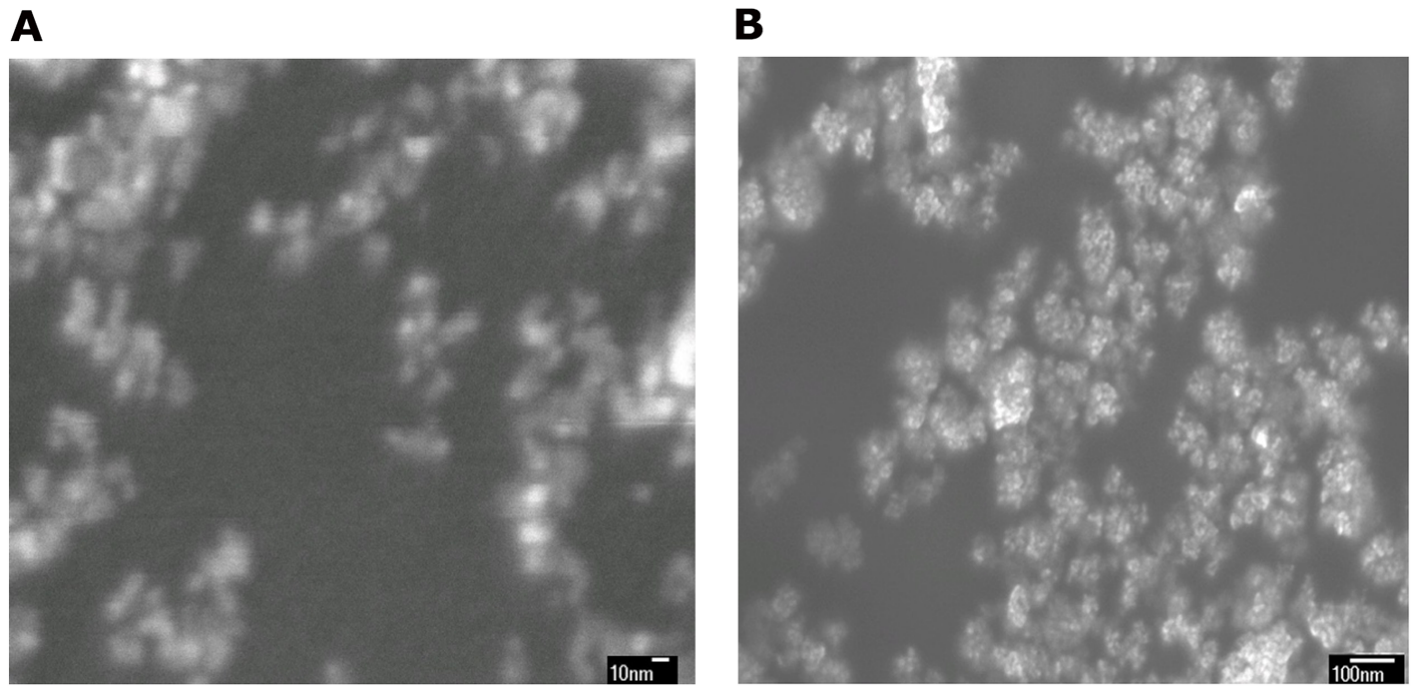
The SEM images of 5–20 nm nanometer powder mixture at different scales.

The nanometer powder mixtures were prepared by doping reaction of In(OH)_3_ and Sn^3+^, where the mole ratio of In and Sn was about 6∶1 as measured by the X-ray fluorescence spectrometer(XRF), The samples were heated at 350°C to form a nanometer powder mixture of In(OH)_3_, In_2_O_3_, and SnO_2_ ((abbreviation: ISOH,some of In_2_O_3_ is Sn^3+^ doped in the In_2_O_3_, it is ITO).). Figure 1 shows spectrum of the powder mixtures by x-ray diffractometer (XRD). The ratio of In_2_O_3_(ITO): In(OH)_3_:SnO_2_ is about 60%∶35%∶5%. The In_2_O_3_ XRD data show that some of Sn^3+^ doped in In_2_O_3_. Figure 2 shows the microstructure features of the nanometer powders using scanning electron microscope (SEM). It can be seen that the micro structural images resemble a nanoscale flower, with a solid core in a size of 5 nm and petal structure in a dimension of 5–10 nm. As a comparison, purity ITO powder is yellow, but ISOH powder is yellowish green.

### 2. Equipments

A scanning electron microscope (SEM, JSM 6700F, JEOL Instrument, made in Japan) was used in this work for microstructure examination.

The microstructure of the nanometer powder mixture was measured by x-ray diffractometer (XRD, D8 ADVANCE, Cu/40kv, 35 mA. BRUKER AXS GMBH, Made in Germany).

The composition of the nanometer powder mixture was measured X-ray fluorescence spectrometer (XRF, S4 EXPLORER, BRUKER AXS GMBH, and Made in Germany).

The optical photos were taken using a Sony digital camera.

The four-column hydraulic press equipment was used to compress the ISOH powers into a shape of disk (made in China).

### 3. Experiment Process

The 5–20 nm flower-like nanometer powders were put into a small mould and pressured to form a disk in a thickness of 1 mm by a small four - column hydraulic press. The disk was tested at different pressures, and its colour change was observed and photographed. The surface of the disk was then examined using SEM, which showed the surface of the disk was made of numerous nanometer voids. The disk strength increases with the pressure improving and heighten decrease (from 10 mm to 1 mm), its strength can change from 0.25 MPa to 0.9 MPa, and it is related to the size of pressing pressure and persisting time.

## Results and Discussion

**Figure 3:**
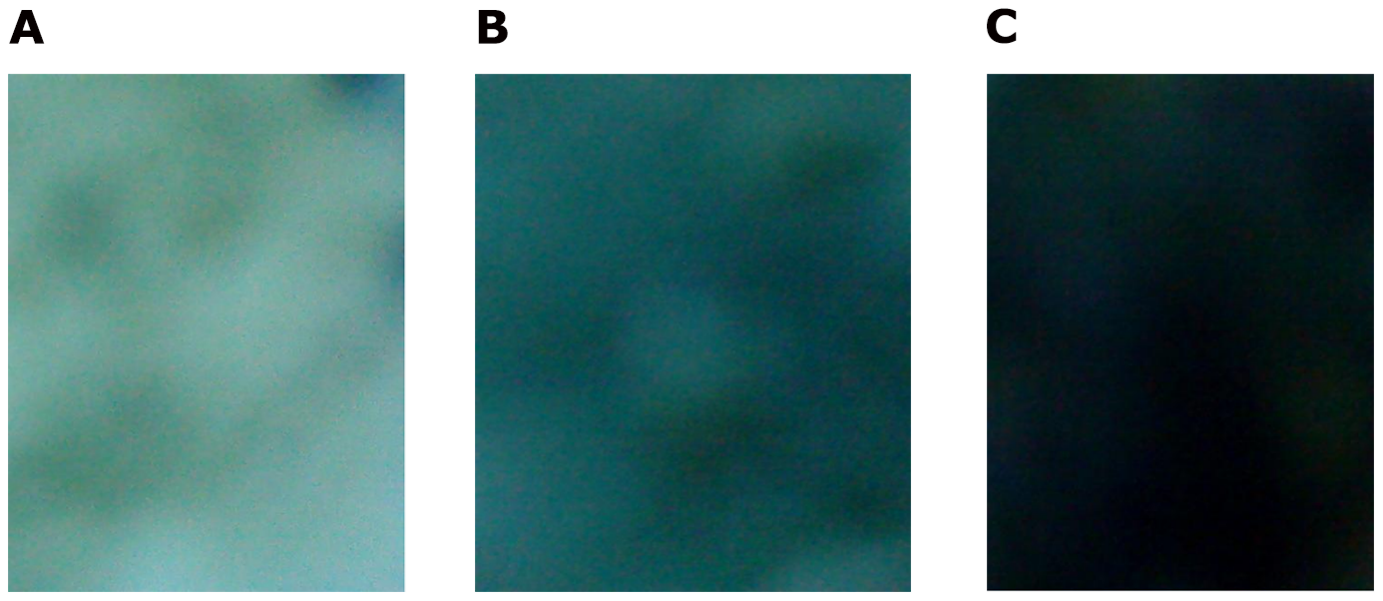
Optical photos showing different colours of the disk under various pressure levels. a) No pressure; b) at a pressure of 0.02 GPa for 60 seconds; and c) at 0.05 GPa for 60 seconds.

**Figure 4:**
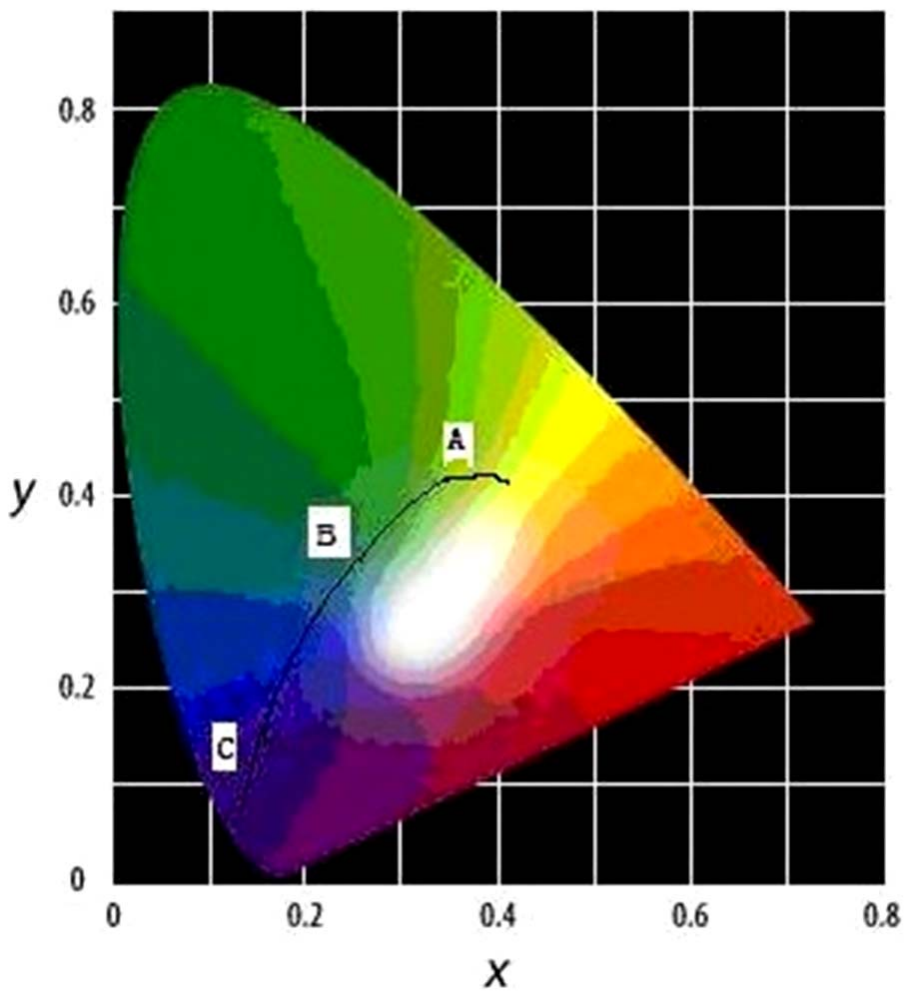
The color change from A–B–C at chromaticity coordinates.

The colour change of the ISOH powders under various pressure levels is a continuous process, as showed in Fig. 3 which was taken by a Sony digital camera. When pressing the powders to form a disk, the colour was changed from an original yellowish green to blue. Further increase in pressure, it then became a colour of blue-black (Fig. 4).

### 1. SEM Examination

**Figure 5:**
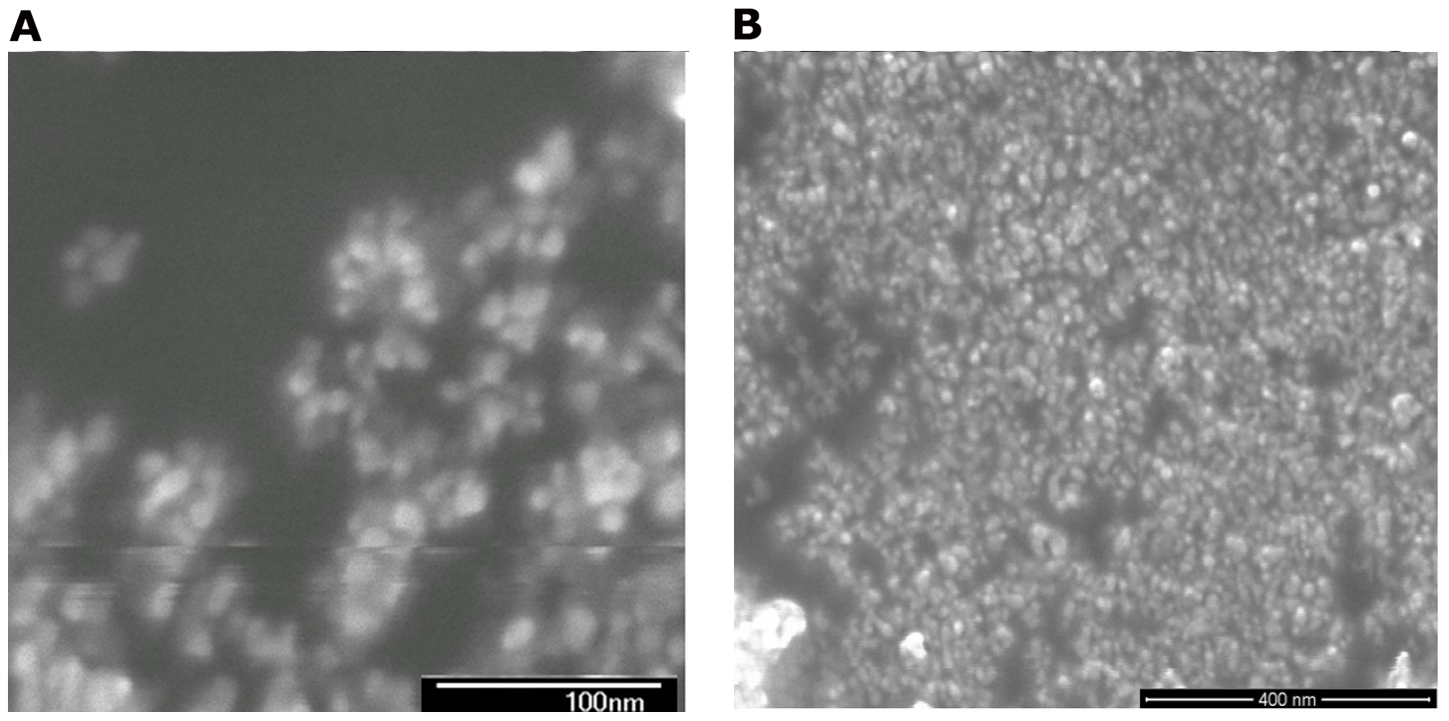
The typical SEM microstructure features of surface of a disk under zero pressure at different magnifications.

**Figure 6:**
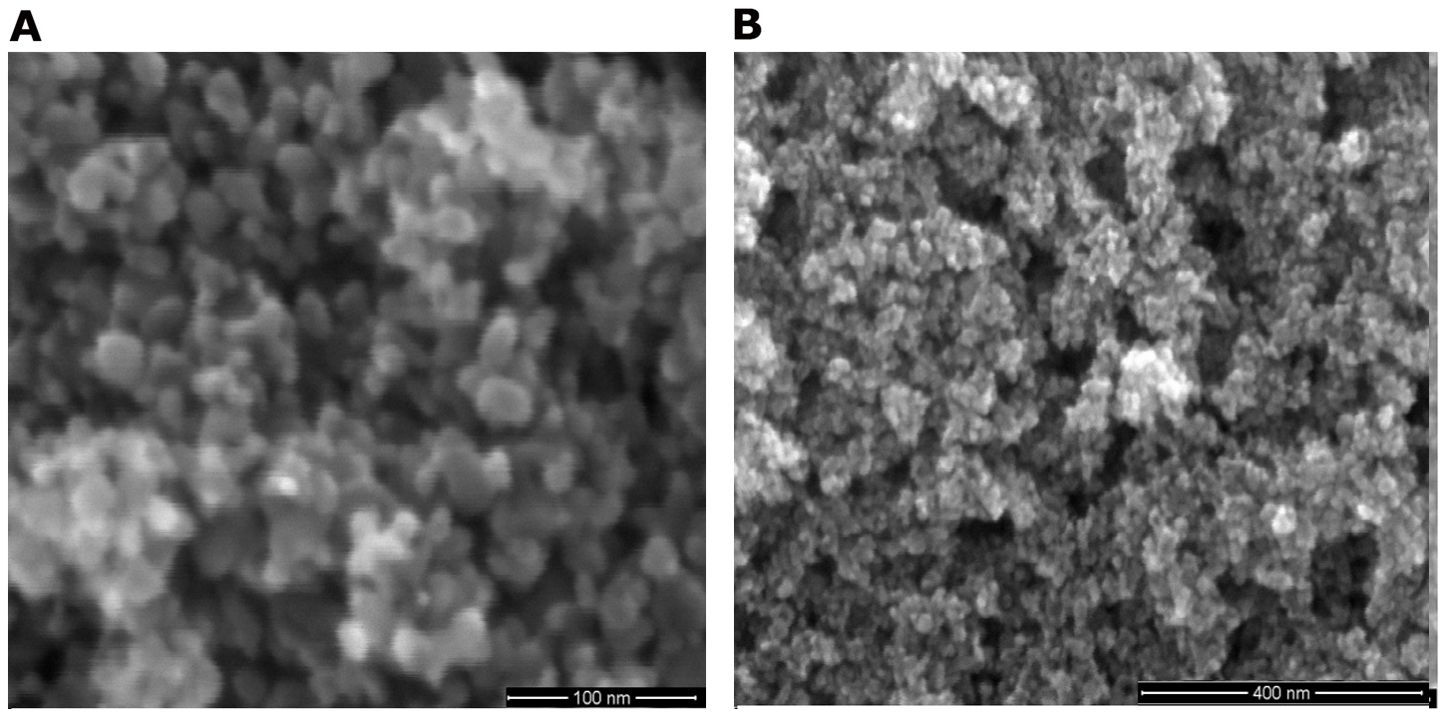
The typical SEM microstructure features of surface of a disk under a pressure of 0.002

The phenomenon of changing the colour of the disk with changing in pressure was also observed using SEM. It was noticed that on the surface of the disks under zero pressure, there are interstices presented between the most particles (>90%), as shown in Fig. 5. When the disk was under a pressure of 0.002 GPa, the interstices vanished among the majority powder particles (>60%). Accordingly many nanoscale voids were formed. These voids were in depths between 5–150 nm, while most of them concentrated in a range of 50–150 nm. Their diameters were in a wide range of 5–200 nm, but with a high percentage within 50–150 nm range. SEM obsevation of the disk surface appearance, as shown in Fig. 6.

**Figure 7:**
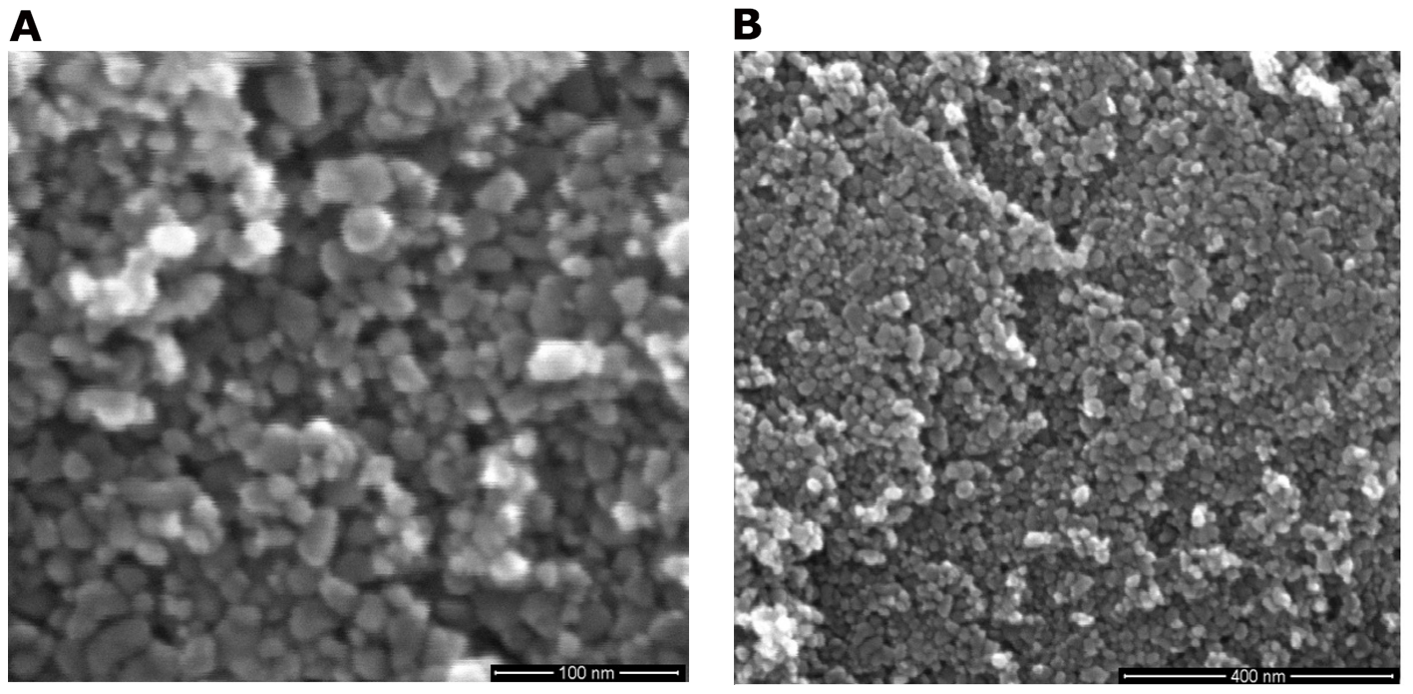
The typical SEM microstructure features of surface of a disk under a pressure of 0.005

**Figure 8:**
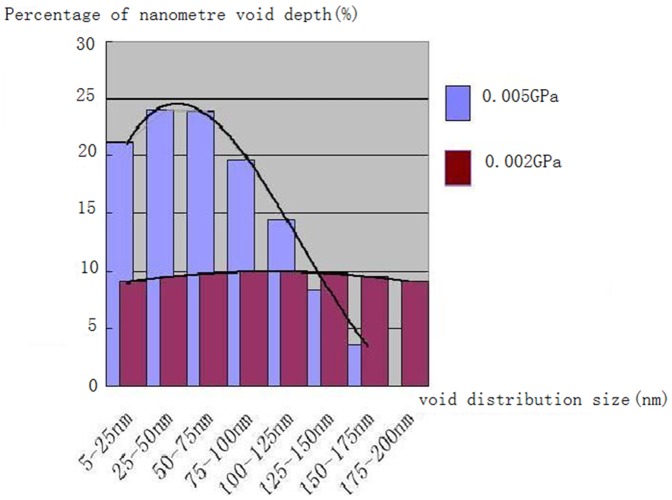
Correlation between size distribution of the nanoscale voids and pressure.

Fig. 7 further reveals that as the pressure was increased to 0.005 GPa, most of the interstices disappeaded and more than 90% powder particles aggregated together to form clusters. Dimensions of the nanoscale voids on the disk surface were also changed. The analysis of the SEM images of Fig. 7 indicats that the pit depths are between 5–100 nm with majority of them in a range of 5–50 nm. As compared with those under a pressure of 0.002 GPa, the depths of the nanoscale voids here decreased. It can also be seen voids' diameters were reduced too, between 5–200 nm. A correlation between size distribution of the nanoscale voids and pressure are summarized in Fig. 8.

It was noticed that the nanometer particles themselves, in terms of dimension and shape, did not change during the loading process. The piezochromic process is reversible. If the disk was grinded back to powders, the powder mixture's colour could change back to yellowish green.

### 2. Effects of Pressure

**Figure 9:**
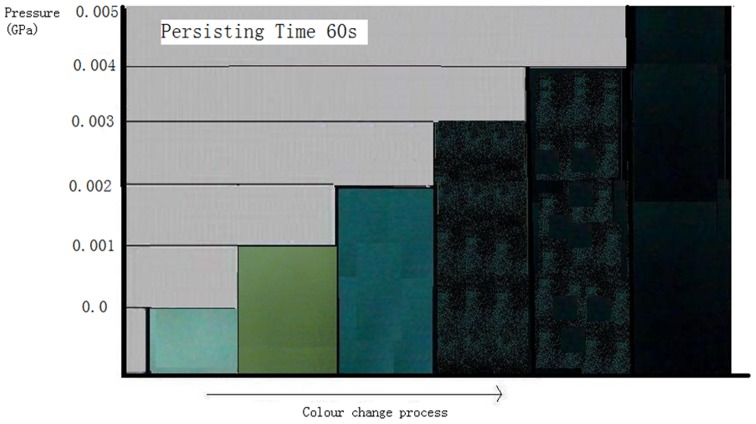
The relation schematic between pressure and colour after 60

The ISOH powders could change their colour, from original yellowish green to blue, in about 60 seconds under 0.002 GPa. With an increase in the pressure level, the speed of piezochromism would increase as well. Under a pressure of 0.005 GPa, the powders could complete the colour change process, from yellowish green to blue, in about 30 seconds and further from blue to blue-black in about another 30 seconds. Fig. 9 shows the relation between pressures and colours after 60 seconds under the loading (Note: The schematic of colour has error, the colour is uneven on the surface of disk, which is not obvious).

### 3. Effects of Holding Time of Pressure

**Figure 10:**
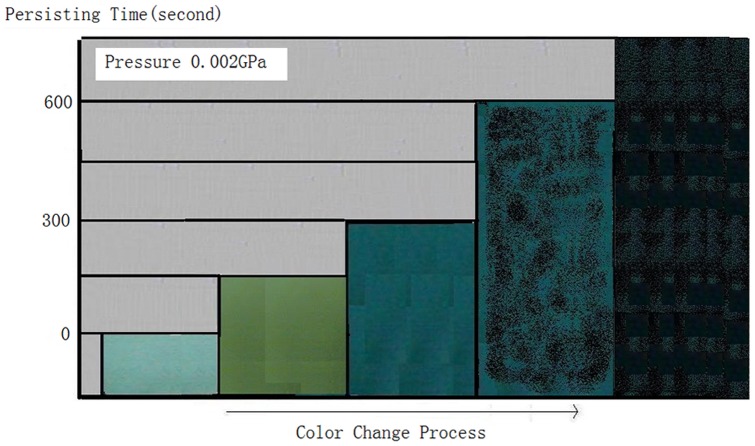
The relation schematic of holding time of pressure and colour change under a constant pressure of 0.002

If the pressure was a constant, such as at 0.002 GPa, while holding time of pressure was prolonged, the colour change from yellowish green to blue would take 300 seconds, then 1200 seconds more to a status of blue-black. Holding time of pressure is the secondary sensitive factor in the piezochromism process, while pressure is the primary one (Fig. 10). The nanometer powder can change colour at more low pressure, but the time prolonged to tens of minutes.

### 4. Effects of Temperature

If the powder mixture was heated to a temperature range of 25 to 80°C, its colour was not changed. When the powders were compressed, its surface temperature could rise by about 5–10°C(including error). The temperature increase is so small that it can only be considered as an auxiliary factor in this piezochromism process. The ISOH nanometer powders are stable below 350°C, which is much higher than the stable temperature of traditional polymer piezochromic materials.

### 5. Analysis of the Results

The colour of a solid depends on several factors, i.e. absorption, reflection, scattering, refraction and diffraction of ray by its surface microstructure. It can be expressed in the following equation:
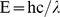
Where: E - energy; h - Planck constant; c - speed of light; and λ - wavelength.

The wavelengths determine the color of a solid. The 5–20 nm ISOH particles used in this work could be dispersed very well and form a uniform nanometer microstructure, quantum confinement effect is obvious inside one 5–20 nm semi-conductive nanometer particle. The diameter of most particles (>90%) is far below vision light wavelength. The number of nanometer particles is 10000–40000 times of 1 micron meter ISOH particles at the same unit area. The phenomena of diffraction, scattering and reflection would be strengthened by such uniform, nanoscale particle size and associated extreme large number of particles. The ISOH flower-like nanometer powders are semiconductor which could absorb some light quantum. The optical band gap width can be affected by the nanometer void different diameter [21] [26], the compacted pressure change the distribution of void's diameter, the surface color of disk change with it. When the ISOH nanometer powders were compressed to a solid shape of disk, nanometer voids formed at its surface, the quantum optical change can be strengthened by the nanometer void also which affected by the quantum confinement [7] [26], it is one factor that strengthen color change, especially nanometer powder has approximately approximate appearance and size; the another factor is surface Plasmon Resonance, ISOH include two ingredients: conductive ITO powder and insulation hydroxide, they have great difference at dielectric property, the distance of two ISOH particle can decrease to only 1–20 nm with the pressure action, when the ray irradiates the ISOH powder surface, these two microstructure particle edges can cause surface Plasmon wave easily, it can lead to dispersion effect, the dispersion effect of light can be diffracted and reflected by the near powder, the pressure change the distance distribution of different particles, it strengthened the surface Plasmon wave with the color change.

The ISOH mono-dispersed nanometer powders' color sensitivity to pressure was increased by tens of times than the normal material, the color change range is wider than the same micrometer powder. If the ISOH powder is agglomerate, not mono-dispersed, or micrometer size, its piezochromic phenomena are not obvious and uniform at low pressure.

As the pressure increased, the size of more nanometer voids became smaller than the wavelength λ of the visible light, thus a larger range of wavelength of the visible rays could be absorbed; the ray dispersion effect that caused by the surface Plasmon Resonance when the two nanometer particle approach 1–20 nm [15]–[21]. The electronic quantum state of surface atom on mono-dispersed ISOH semi-conduct particle change with the nanometer distance between them. The color of the disk surface could be changed [21]–[26]. In addition, the piezochromic process is generally gradual and continuing.

## Conclusions

The color of inorganic ISOH nanometer powders was sensitive to compacting pressure. The piezochromic process could be initiated in this material at a low pressure level of 0.002–0.01 GPa. As compared with traditional piezochromic materials, its sensitivity to pressure was increased by tens of times, and the color change process was reversible below 350°C, the performance of piezochromic is very stable between −20°C to 150°C. The ISOH nanometer powders will have great potential applications in various pressure detective equipment and processes. For example, it will be especially useful in the long-range forecast of the geological hazards.
